# Gender Medicine and Physiotherapy: A Need for Education. Findings from an Italian National Survey

**DOI:** 10.3390/healthcare8040516

**Published:** 2020-11-27

**Authors:** Mattia Bisconti, Fabrizio Brindisino, Filippo Maselli

**Affiliations:** 1 Department of Medicine and Health Science “Vincenzo Tiberio”, University of Molise, c/o Cardarelli Hospital, C/da Tappino, 86100 Campobasso, Italy; mattia.bisconti@unimol.it (M.B.); fabrizio.brindisino@unimol.it (F.B.); 2 Physiotherapy and Manual Therapy, Physiotherapy Department UPMC Italy Salvator Mundi International Hospital, Viale delle Mura Gianicolensi, 67, 00152 Rome, Italy; 3 Faculty of Medicine, University “Tor Vergata”, Via Montpellier, 1, 00133 Rome, Italy; 4 Department of Neurosciences, Rehabilitation, Ophthalmology, Genetic and Maternal Infantile Sciences (DINOGMI), University of Genova—Campus of Savona, 17100 Savona, Italy; 5 Sovrintendenza Sanitaria Regionale Puglia INAIL, 70126 Bari, Italy

**Keywords:** gender medicine, physiotherapy, knowledge, physiotherapy’ clinical practice, beliefs

## Abstract

Background: Gender medicine permeates all sectors of medicine, from prevention to treatment and rehabilitation; it aims to customize the care path, ensuring equity in the access to health care system services. It is unclear to what extent physiotherapists’ treatment choices align with gender medicine principles. The aim of this survey is to detect the need of Italian physiotherapists to deepen knowledge in gender medicine. Materials and methods: An 18-item survey assessed the characteristics of responders about knowledge of gender medicine. An online survey was performed in 2020 using SurveyMonkey Software. Data were analyzed by statistical regression. Results: A total of 617 physiotherapists voluntarily participated in the study (53.84% of the target population). The majority of responders (68.4%) declared having general information about gender medicine, but 55.43% of them claimed to have heard of it but did not know properly what gender medicine was about; 92.38% of the physiotherapists believed that they needed training to acquire knowledge in gender medicine. Conclusions: Gender equity matters for health. Moderate knowledge of gender medicine’ principles and modest application of these findings were used in clinical practice of physiotherapy. Physiotherapists declared that they need training in gender medicine.

## 1. Introduction

### 1.1. Field and Background

The fields of science, medicine, and global health are in the midst of a gender reckoning [1]. The purpose of gender medicine (GM) is to ensure equity in health care and access to care: on the one hand it is a question of understanding whether men, women and gender-diverse people should be treated differently, on the other it represents a way to study people, not only biologically, but in a more complete and complex way, referring to gender because restrictive gender norms affect everybody. As a shared determinant of health, gender inequalities drive large-scale excesses in mortality and morbidity globally [2]. The gender approach to health is a cultural process that takes time to overcome stereotypes, prejudices, inequalities in research, and in the study of the causes and risk factors for a given disease. Deriving from the complaint that medicine is not a neutral science, it has become increasingly surprising to see how the development of medicine had taken place through studies conducted almost only on men, based on the scientific, incorrect prejudice, that the women’s body is like that of the men, with the only differences referring to the sexual and procreative apparatus [3]. The term gender is a “widely” used and often misunderstood term: it is sometimes mistakenly conflicted with sex or used to refer only to women [2], and also categorically excludes transgender and non-gender binary people [2,4].

The concepts of sex and gender are often improperly superimposed. In this study, by “sex” we mean the different biological and physiological characteristics between males and females (i.e., chromosomes, hormones), with the term “gender” we refer to a social construction of norms, prescriptions, activities, relationships and attributes of a data society considered appropriate for men, women and gender-diverse people, referring to the specific historical moment and to one’s own culture, based on social typologies [5].

We cannot expect the mass diffusion of the correct nomenclature of “sex” and “gender” through the media, people and health care professional if the definitive change is not first internalized and disseminated by scientific publications [6,7]. The World Health Organization (WHO) focuses attention on the gender dimension, indicating how indispensable the development of research in the area of medicine is for inequities [8]. The WHO has developed a Gender Policy to ensure that all research programs and projects are structured with a gender perspective and this is done in a sustainable and systematic way [9]. Since 2018, in Italy it has been guaranteed that medicine is gender oriented in all its applications (and, therefore, also in physiotherapy) [10]. 

There is widespread consensus that gender equality in science, medicine, and global health has the potential to lead to substantial health, social, and economic gains [11,12].

Physiotherapists during their clinical practice or during research activities are not immune from gender influences and from all the variables related to it. The stereotyped component of the concepts of masculinity and femininity has been strongly emphasized as verifiable, which influences the treatment of the person according to gender [9,12,13,14,15]. For example, the differences that exist between men and women in perception, expression and pain tolerance are influenced by a wide variety of social and psychological processes [12]. Gender roles have been associated with pain response in a stereotyped way: the male gender “normally” has higher pain tolerance, while the rules governing female gender behavior impose pain acceptance as a normal part of life and would lead women to be more permissive to themselves in the expression of pain [12,16,17,18]. It turns out that a “stoic man” would be more inclined to avoid talking about his painful state both as a tool to maintain his status of virility, and as a phenomenon of denial of a state of weakness more likely to be attributed to the female gender [9,14,19]. Women, on the other side, well-taught and more skilled to talk about their pain, would tend to seek greater social support to better manage their pain and it has been reported how this strategy can be beneficial for the outcome of pain and return to work [13]. Cultural background and level of education [9,13,14,16,20], social role and socio-economic status [9,13,15,20,21,22] influence pain modulation and are considered to influence gender. All modern societies have a gender order built from ideas about what is seen male and female, feminine and masculine, in which male domination and masculinity is created and maintained [3,23]. 

Recognizing the differences related to the socio-cultural dimension of gender is useful for outlining activities and programs to organize the offer of services, to direct research and to analyze statistical data: it is essential to connect the GM dimension to methodological need, analytical and system governance tools. Along with the spending reviews adopted by governments, it would be appropriate to add the application of a medicine that takes into account sexual and gender differences to improve the health environment, also bringing a reduction of costs with savings estimated in Italy of around €100 million [24]. 

Consequently, not only a health environment based on equity would be obtained, but economic benefits would also derive for the health system itself, according to the principles of appropriateness of care [24].

### 1.2. Policy and Reason of the Study

In science, the division in knowledge between genders continues to exist in all countries, even those that have a highly developed knowledge society [2,25]. In Italy, on 31 January 2018, the “Gazzetta Ufficiale” no. 3/2018 published the “Application and diffusion of gender medicine in the national health system” [24]. In two specific points it is possible to find highlights on professional training and professional courses, communication and information (i.e., inform and sensitize health workers and researchers on the importance of a gender approach in every area of medicine) [26].

### 1.3. Training in Gender Medicine

In the decree of the Italian Minister of the University and of scientific and technological research of 5 October 2000, published in the ordinary supplement of the “Gazzetta Ufficiale” no. 249 of 24 October 2000, the gender sensitive perspective is placed among the educational objectives in six classes of three-year degree courses out of 26 (including physical and sport sciences) and in 11 classes out of 52 of master’s degree (including medicine and surgery and sports and sports sciences). From the data referring to the academic year 2011/2012, on a sample of 57 Italian public universities, it was found that only 16 universities had activated courses on gender (0.001% of the entire Italian university educational offer, 20% of which in the medicine area) [26].

### 1.4. Objective

In adherence to the Italian legislative plan, law 3/2018 art.3 [24,26]: “application and diffusion of gender medicine in the national health system” [10,24], the aim of the questionnaire is to: (a) acquire descriptive information on the sample, (b) acquire information on the basic knowledge of the sample about of gender medicine (c) detect the need of the physiotherapist to deepen knowledge on gender medicine, through dedicated education and training courses.

## 2. Materials and Methods 

### 2.1. Design

A quantitative exploratory web-based cross-sectional survey was performed according to the Checklist for Reporting Results of Internet E-Survey (CHERRIES) guidelines [27] and Strengthening the Reporting of Observational Studies in Epidemiology (STROBE) [28]. Survey was administered in Italy between July and September 2020. This study has approved by the Ethics Committee of “Azienda Sanitaria Locale (ASL) of Lecce” (Italy), (protocol number 53, 30 September 2020).

### 2.2. Participants and Setting

A nationwide sample of Italian physiotherapists or students enrolled in the third year of the degree course in physiotherapy subscribed to the GTM (Manual Therapy and Musculoskeletal physiotherapy group) mailing list was the target population (*n* = 1146). The GTM, IFOMPT (International Federation of Orthopaedic Manipulative Physical Therapists) representative in Italy [29], represents a point of reference for professionals who want to pursue an advanced training course in physiotherapy. Within the established target population, we included those physiotherapists who: (a) had a valid e-mail account (b) were GTM associated, (c) understood the Italian language. A sample size of 617 people setting an interval confidence at 95% with a 5% error margin was calculated considering the number of Italian physiotherapists present in the GTM register.

### 2.3. Questionnaire Developments and Pre-Testing

A survey tool was developed composed of questions using distinct and interactive steps [30]. Items from existing survey on gender medicine in nursing were extracted [31].

The initial list included 20 questions that were evaluated for face and content validity [30] by a panel of 7 expert OMPT (Orthopaedic Manipulative Physical Therapist) physiotherapists, 1 Medical Doctor, 1 Nurse and 1 Psychotherapist with extensive experience in survey design and GM. These experts worked independently and then agreed on the final list providing feedback on content accuracy, wording, question order and survey structure. Considering the feedback that emerged, adjustments were included. After reaching full agreement among experts, a preliminary version of the survey composed of 18 questions was self-administered and piloted in a convenient sample of 15 physiotherapists. Once the pilot stage was over, a telephone debriefing session was performed [30]. The panel of experts conducted one-to-one interviews among the sample of 15 physiotherapists on the possible difficulties encountered answering the survey (e.g., identifying questions that required further explanation and the physiotherapists experience in answering the questions). The sample reported mainly that questions were not ambiguous, wording was easy and simple to be understood and the self-administered experience was successful.

### 2.4. Questionnaire Implementation

It was used a self-administered questionnaire (English version in Supplementary Materials) divided into 3 sections (A, B and C): in section A, the socio-demographic variables were investigated by 5 closed-ended questions (i.e., sex—to identify the biological sex of participants, educational level); in section B knowledge on gender medicine was investigated by 8 closed-ended questions (the questions probe the sample in order to find out whether the respondents have background knowledge on gender medicine); in section C training already completed and desire for training in GM for physiotherapists were investigated by 5 closed-ended questions. The possible answers, which can be selected through a list of check-boxes shown to the respondents, were decided by the study authors, then modified and confirmed by the authors and expert panels during the survey structuring phases.

### 2.5. Data Collection Procedure

It was used the SurveyMonkey online survey software [32]. The survey was administered between July and September 2020. After GTM permission, all mailing list subscribers were contacted by e-mail [30] containing the link to the survey and a brief note outlining the aim of the study, data handling (anonymity), informed consent statement, invitation to complete the survey, presentation of the authors; by clicking on the survey link, respondents provided their consent to participate [30].

Participation was voluntary and no incentives were offered to participants, all questions were compulsory, it was possible to quit the questionnaire at any time [30]. Participants were able to review or change the responses using a back button before submitting their answers.

Data were downloaded and stored in an encrypted computer and only the authors had access to the information during all stages of the study. Participants were ensured that their identities would not be disclosed to investigators: all data were de-identified to maintain confidentiality and data protection [30]. An e-mail reminder was sent 2 weeks after the initial contact [30].

### 2.6. Data Analysis

The presence of any relationship between the individual’s characteristics and the responses given was investigated with the econometric software “Stata”, applying a Logit-type regression, with a *p* < 0.05 considered significant.

The model specification is as follows “〖PFG〗_i = α + β Xi + ε i” where:

- “〖PFG〗_i is a dummy variable that assumes a value of 1 for subjects who in their clinical activity apply the principles of gender medicine in physiotherapy, value 0 in the opposite case;
- “α“ represents the constant;
- “β“ measures the relationship between the control variables and the dependent variable;
- “Xi” vector containing all the variables that can influence the probability that the physiotherapist applies the principles of gender medicine in physiotherapy;
- “ε i” represents the error term.

The control variables contained in the vector X_i are the following: age, sex, years of work, type of University degree, type of institution where the practitioner works (i.e., public or private), physiotherapists who have heard about GM, physiotherapists who claim to know what GM is about, physiotherapists who are able to recognize the correct definition of GM, physiotherapists who participated in training courses or information events on GM, physiotherapists who participated in training courses or information events in the field of GM specific for physiotherapists, physiotherapists who believe that their profession requires courses or training events to acquire deeper knowledge on GM.

The empirical analysis is based on survey data downloaded from SurveyMonkey [32] into Excel spreadsheets and reviewed for accuracy and missing value. A questionnaire was considered incomplete and was not included in the calculation of valid answers if the participant quit the compilation [33].

All questions allowed only one choice and provides descriptive statistics. Ages and years of clinical experiences were transformed into ordinal variables. 

## 3. Results

### 3.1. Participant’s Characteristics

Out of 1146 addresses, a total of 617 responded (53.84%). No incomplete questionnaire was recorded, all of the 617 responses were considered valid for the analysis; moreover in case of incomplete answer the survey would have been eliminated, hence no questionnaire was left uncompleted. The 45% of respondents were men (*n* = 281; SD = 0.5); 43.76% were between 30 and 39 years (*n* = 270, SD = 0.96); 57.7% of participants had worked as physiotherapists from less than 10 years (*n* = 356, SD = 1.03); 54.95% had a postgraduate degree (*n* = 339, SD = 0.5); and 74.39% of participants (*n* = 459, SD = 0.44) practiced in a private institution (i.e., private practice, private clinic). All these described variables were inserted into the econometric model and are shown in Table 1.

### 3.2. Knowledge about Gender Medicine

The majority of responders (68.4%, *n* = 422) declared having general information about GM, specifically only 12.97% (*n* = 80) declared knowing what GM is, 55.43% (*n*= 342) claimed to have only heard of it but did not know what GM is; 31.6% (*n* = 195) replied saying they had never heard of GM; 35.01% (*n* = 401, SD = 0.48) said they know what GM is. The majority of the physiotherapists interviewed (55.92%, *n* = 345, SD = 0.5), managed to identify the correct definition among the proposals for GM, the other part (44.08%, *n* = 272) responded differently, specifically 9.72% (*n* = 60) responded that GM is an approach that aims to eliminate health inequalities; 6.48% (*n* = 40) answered that there was no correct answer among those listed; the 27.88% (*n* = 172) responded to the question by selecting the answer similar to the correct one but which showed an inversion of the definition of the words “sex” and “gender”. All these described variables were inserted into the econometric model and are shown in Table 2.

It was possible to identify the characteristics of the participants (age, sex, degree) who did not provide the correct definition of GM as shown in Table 3: they were almost uniformly distributed by age group. Subjects <29 years of age were less mistaken (37%) than physiotherapists who were between 30 and 39 years old (46%), 40–49 years old (56%), >50 years old (45%). Male and female responders who could not identify the correct answer on the definition of GM had an almost overlapping percentage: 43% for female and 45% for male. Specifically, the right answer was: Gender medicine poses a different and innovative approach to health inequities, linked not only to a different diagnostic-prescriptive appropriateness, but also to social, cultural and ethnic, psychological, economic and political inequities. This distinguishes the concept of gender (a person’s way of seeing himself as male and female with respect to his social role) from that of sex (relating to reproductive functions and biological differences); and the wrong answer provided only an inversion of the terms “sex” and “gender”. The sample (39%) that had the level of education identifiable as a first-level postgraduate university degree was unable to identify the correct definition of GM. Students enrolled in the 3rd year of the degree course in physiotherapy and holders of a PhD, with a percentage respectively of 64% and 63%, gave the wrong answer. 

### 3.3. Training in Gender Medicine

The minority of the responders (43.27%, *n* = 267, SD = 0.5) applied the principles of GM in physiotherapy, only 6.65% (*n* = 41, SD = 0.25) of responders had participated in training courses in GM and only 4.05% (*n* = 25, SD = 0.19) had participated in training courses in specific training in GM for physiotherapists. Despite the lack of participation in training courses, 92.38% of the physiotherapists interviewed (*n* = 570) believed that their profession needs courses or events to acquire deeper knowledge in GM. All the described variables were inserted into the econometric model in Table 4.

### 3.4. Statistical Regression

The statistical regression results are highlighted in Table 5 and show that sex is a highly significant variable capable of influencing the choice of physiotherapists to apply the principles of GM in physiotherapy: men are more likely to apply the principles of GM in physiotherapy (odds ratio (OR) = 1.43; 95% confidence interval (CI) 1.01–2.05; *p* < 0.05). Working mainly in a private entity significantly increases the probability of applying the principles of GM in physiotherapy (OR = 2.36; 95%CI 1.51–3.69; *p* < 0.01).

Furthermore, the probability of applying the principles of GM in physiotherapy increased significantly for physiotherapists who claimed to know what GM is (OR = 2.67; 95% CI 1.75–4.07; *p* < 0.01), are able to identify the correct definition of GM (OR = 1.37; 95%CI 0.97–1.94; *p* < 0.1), or believe that their profession requires courses or events to acquire more knowledge on GM (OR = 2.36; 95%CI 1.14–4.9; *p* < 0.05).

Finally, the probability of applying the principles of GM in physiotherapy was not significantly influenced by age, years of activity as a physiotherapist, type of training received (degree), having heard of GM or participated in training courses or events in the field of GM, and GM for physiotherapists.

## 4. Discussion

To the best of our knowledge, this is the first study that evaluates awareness on GM among physiotherapists. The highly significant variables for “Application of the principles of Gender Medicine” are sex (*p* < 0.05) and institution where the physiotherapists work (*p* < 0.01). Those who are men are more likely to respond to apply the principles of GM in physiotherapy; in addition those who work in an institution or a private practice are more likely to respond to adopt the principles of GM in physiotherapy: due to greater attention to contextual factors (CFs) as described in the ICF (International Classification of Functioning, Disability and Health) document [34], it is probably easier to create the optimal condition for a good therapeutic relationship and for clinical improvement.

The probability of applying the principles of GM in physiotherapy increases significantly (*p* < 0.01) for physiotherapists who declare that they know what GM is concerned with (OR = 2.67): it may be useful to proceed to a greater diffusion and implementation of training courses on the theme of GM. Physiotherapists who can identify the correct definition of GM among those proposed are more likely to adopt the principles of GM (OR = 1.37), but the effect is scarcely significant (*p* < 0.1). The probability of applying the principles of GM increases significantly (*p* < 0.05) for physiotherapists who consider training courses necessary to acquire more knowledge on GM (OR = 2.36): specifically, 92.38% (45.06% male, 54.73% female) of physiotherapists believe that training courses are necessary to acquire knowledge on GM; by contrast, 7.62% (48.93% females, 51.06% males) of physiotherapists do not consider training courses on GM necessary. We can therefore hypothesize that healthcare personnel who are already committed to equity in care path are more willing to deepen their knowledge in GM. Application of GM in physiotherapy in Italy is not uniform and still depends on the single healthcare professional, because of the lack of GM based courses in many Italian universities, the complex and transversal nature of the topic, still seen by many professionals as new and innovative.

The use of correct terminology can show a step towards greater attention in the appropriate treatment of each patient: “sex” and “gender” were the terms investigated in detail of the definition precisely in order to identify the percentage of physiotherapists who are confident of the difference and to understand which starting point training activities could have.

Treatment of patients is probably gender-biased because clinicians are influenced by the higher status of men and the power relationships that are unfair within society itself. For women, their responsibility towards family and children was seen as an obstacle to recovery and return to work [9]. Organizations such as families, workplaces, health care and social security systems are structured by dominant gender relations in a specific society, therefore expectations about social role could influence results of rehabilitation in different ways for women and men [35]. We see how health workers advise women to give up work more often, or advise less physical activity and more drug therapy [13,36,37]. Furthermore, widening our perspective, there is evidence that different patients prefer to be treated by a specific gendered doctor [38], which is important for equity of access to care.

A Canadian study concluded that gender is a marker of other behaviors that lead to better outcomes, pointing to evidence that female doctors tend to follow guidelines more closely, spend more time with patients, and might have more effective communication skills than male doctors [39]. In one meta-analysis of the gender effect in medical communication [40], female primary care physicians had a more patient centered communication style; however, there was no gender difference in the quality of information conveyed to patients, and male obstetrics and gynecology specialists scored higher for emotionally focused talk than did female specialists. The drivers of these observed differences should be investigated to elucidate the positive behaviors that lead to outcomes to optimize training and development for the entire science and health workforce [2].

Studies describing the behavior of health workers dedicated to rehabilitation and physiotherapy were conducted in Sweden [9,20]. As clinicians, we tend to take better care of patients who behave like a man or woman who meet role expectations. Regardless of whether or not they have similar needs, patients who are white men, educated, well-dressed are believed to be more job-oriented, flexible and with cognitive abilities that encourage them in the rehabilitation process and these notions are confirmed by the perceptions of the patients [9]. Knowing these components can be useful for the physiotherapist to modulate his clinical intervention in a more appropriate and effective way and in compliance with the bio-psycho-social model of medicine. Gender inequity is transformed into health risk through the following process: discriminatory norms, beliefs, and practices; differential exposures and susceptibilities to disease, disability, and injuries; biases in health systems and health research [2].

### 4.1. Strengths and Weaknesses of the Study

To date, no other surveys have been conducted that investigated the training needs for the physiotherapists in Italy in GM: for this reason, it is not possible to indicate whether the response rate achieved here (53.84%) is satisfactory. The answers given by physiotherapists could be influenced in the direction of overestimation compared to the average by their degree of specialization. A survey tool was adopted to understand the perspectives of the target population [41]. The survey included different items to increase the likelihood of capturing the complexity of the phenomena under study [42]. Since the data were self-reported and retrospective in nature, distortion of memory can threaten the validity of the results [43,44]. Despite the certainty of anonymity, some participants may have erroneously reported their knowledge of gender medicine. The survey was based on the analysis of the articles found in the literature regarding the improper use of the terms “sex” and “gender” [6] and, the knowledge and application of the principles of GM by health professionals [9,20,21,40,41].

### 4.2. Limitations

Considering that the data collected were self-reported and retrospective, recall and response biases may limit the internal and external validity of the findings. It is desirable to select a larger sample that includes a greater number of Italian physiotherapists. The main limit to the study is represented by the absence of quantitative analysis of the survey.

## 5. Conclusions

The concept of gender is an “ongoing process” that points to its roots in time and in the cultural space of derivation, influenced by and influencing society. GM and adequate training of health professionals can be a lever for internal change of society because professionals able to consider the patient in his bio-psycho-social complexity, not spoiled by preconceptions, are the first to be able to undertake training and provide information. We want to improve the concept of equality with that of equity in order to enhance the differences free from preconceptions and gender constraints.

The survey led to highlight how modest the knowledge is in physiotherapy of the principles of GM and instead, how professionals are curious on the subject, explaining the will and need to deepen the study through dedicated training courses. Further quantitative studies are needed in order to evaluate knowledge, uses and attitudes on GM in physiotherapists, between those who work in hospital and in a private practice. To develop a more comprehensive understanding of the clinical dynamics that occur between patient and physiotherapist, there is also a need to investigate patients’ perception of the clinician’s behavior as well as the clinicians’ behavior. Achieving gender equity is not simply instrumental for health and development, its impact has wide-ranging benefits and is a matter of fairness and social justice for everyone.

## Figures and Tables

**Table 1:** Descriptive statistics of the participants’ characteristics.

Participants’ Characteristics	Average	Std. Dev.	Min	Max
Age	2	0.96	1	4
Sex	0.45	0.5	0	1
Years of experience	2.31	1.03	1	4
Degree	0.55	0.5	0	1
Institution	0.74	0.44	0	1

Sex value: 1 for male; Age value: 1 for <29 years, 2 between 30 and 39 years, 3 between 40 and 49 years, value 4 for >50 years; Years of experience value: 1 for <5 years, 2 <10 years, 3 between 10 and 20 years, 4 for >20 years.

**Table 2:** Descriptive statistics for knowledge about gender medicine.

Knowledge about Gender Medicine	Average	Std. Dev.	Min	Max
General information on GM	0.68	0.47	0	1
Knowledge on GM	0.35	0.48	0	1
Definition of GM	0.56	0.5	0	1

Acronyms: GM, gender medicine.

**Table 3:** Wrong definitions for gender medicine and participants’ characteristics.

Responders for Wrong Definition about Gender Medicine	Percentage
**Age**	
<29	37%
30–39	46%
40–49	56%
>50	45%
**Sex**	
Female	43%
Male	45%
**Degree**	
Last year students of Physiotherapy at University	64%
Physiotherapy degree	48%
I level postgraduate University course	39%
II level postgraduate University course	40%
Master of Science	42%
PhD	63%
Total	272

**Table 4:** Training in gender medicine.

Training in Gender Medicine	Media	Std. Dev.	Min	Max
Apply GM in PT	0.43	0.5	0	1
Courses in GM	0.07	0.25	0	1
Courses in GM for PT	0.04	0.19	0	1
Need for training in GM	0.92	0.27	0	1

Acronyms: GM, gender medicine; PT, physiotherapy.

**Table 5:** Statistical regression—factors influencing the application of gender medicine principles.

Factors Influencing	O.R.	*p*-Value	IC 95%	
			Lower	Upper
Age	1.17	0.39	0.82	1.64
Sex *	1.43	0.04	1.01	2.05
Years of experience	1.18	0.31	0.86	1.63
Degree	0.99	0.97	0.69	1.41
Institution *	2.36	0.00	1.51	3.69
General information on GM	1.24	0.31	0.81	1.92
Knowledge on GM *	2.67	0.00	1.75	4.07
Definition on GM	1.37	0.08	0.97	1.94
Courses in GM	1.19	0.67	0.54	2.61
Courses on GM for PT	2.02	0.17	0.74	5.51
Need For training in GM *	2.36	0.02	1.14	4.90

Acronyms: GM, gender medicine; PT, physiotherapy * Statistically significant (*p*-value < 0.05) or approaching significance.

## Supplementary Materials

The following are available online at https://www.mdpi.com/2227-9032/8/4/516/s1. All data generated or analyzed during this study are included in this published study. Other information of this study is available from the corresponding author on reasonable request. Supplementary: Self-administered questionnaire: English version.
