# Supplementary material for: Gender Medicine and Physiotherapy: A Need for Education. Findings from an Italian National Survey
Source: Healthcare (Basel). 2020 Nov 27;8(4):516. doi: 10.3390/healthcare8040516 (PMC7711877; doi:10.3390/healthcare8040516)
Supplement: Supplementary file 1 [file healthcare-08-00516-s001.pdf]

Gender Medicine and Physiotherapy: A Need for Education. Findings from an Italian National Survey

Dear colleague, I'm Mattia Bisconti, OMPT Physiotherapist. I kindly ask you to fill in the following questionnaire. The survey will help me complete my study project. The subject of this investigation is the knowledge of gender medicine by the physiotherapist in Italy. The purpose of the questionnaire is to identify the physiotherapist's need for training in Italy in the field of personal care according to the principles of gender medicine. It takes 5-8 minutes to complete the questionnaire. Thank you in advance for taking the time to complete the questionnaire.

Only one correct answer can be selected.

Your responses will be completely anonymous and will be used solely for the purposes of this research. Participation in the study is on a voluntary basis. All data collected will be processed and stored in a strictly anonymous manner pursuant to Legislative Decree 196/2003 and subsequent amendments and additions regarding the processing of personal data. Only the personnel involved in the study will have access to this information. Access to such data will be protected by the investigator. Your participation will guarantee total confidentiality. Your consent to participate in this study is in fact granted by continuing to fill in the questionnaire. Your consent can be denied without any prejudice, simply by not continuing to fill in the questionnaire.

By continuing to fill in the survey, you in fact grant your consent by declaring: that you have received full explanations regarding the request to participate in the survey as reported in the information premise; that you have received satisfactory answers to all the questions you have deemed necessary; you have been informed of your right to withdraw at any time; that you are at least 18 years old.

---

1. Age
   - < 29 years old
   - 30-39 years old
   - 40-49 years old
   - > 50 years old
2. Sex
   - Female
   - Male
3. How many years have you been a physiotherapist?
   - < 5 years
   - < 10 years
   - 10-20 years
   - > 20 years
4. Current university degree. Select the highest grade, from the categories ordered below (enter the highest academic title)
   - Student enrolled in the last year of the Degree course in Physiotherapy
   - Bachelor's Degree in Physiotherapy or equivalent qualification
   - First level University Master
   - Second level University Master
   - Master of Science
   - PhD

5. Where do you work most of the time?
    - Public service (Hospital)
    - Private service (private practice, private center or private clinic)
    - Sports club/association/federation
    - University (Didactic or research activity)
6. Have you ever heard about Gender Medicine?
    - Yes, I know what Gender Medicine is all about
    - Yes, I've heard of it but I'm not sure what Gender Medicine is about
    - No, I've never heard of it
7. Do you know what gender medicine does?
    - Yes, I know what Gender Medicine does
    - No, I don't know what gender medicine does
8. What do you think could be the best definition of Gender Medicine among those listed below?
    - Gender Medicine poses a different and innovative approach to health inequities, linked not only to a different diagnostic-prescriptive appropriateness, but also to social, cultural and ethnic, psychological, economic and political inequities. Distinguishes the concept of gender (a person's way of seeing himself as male and female with respect to his social role) from that of sex (relating to reproductive functions and biological differences)
    - Gender Medicine poses an approach that aims to eliminate health inequalities, linked not only to a different diagnostic-prescriptive appropriateness, but also to inequalities related to one's sex (relating to reproductive functions and biological differences)
    - Gender Medicine poses a different and innovative approach to health inequities, linked not only to a different diagnostic-prescriptive appropriateness, but also to social, cultural and ethnic, psychological, economic and political inequities. Distinguishes the concept of sex (a person's way of seeing himself as male and female with respect to his social role) from that of gender (relating to reproductive functions and biological differences)
    - It is not among these listed
9. Do you think there is a "gender physiotherapy"?
    - Yes
    - No
10. Do you think it is important to know the "gender differences" in physiotherapy practice?
    - Yes, I think it is important to appropriately modulate assessment strategies and physiotherapy treatment
    - Yes, I think it is important to standardize the treatments
    - Yes, I think it is important to provide all patients with the same type of assessment and treatment, without any difference
    - No, it is not important
11. In clinical practice, do you think a different approach is important according to gender?
    - I do not know
    - No
    - Yes
12. Could a correct application of the principles of gender medicine in physiotherapy affect the patient's prognosis?
    - Yes, because men heal faster than women
    - Yes, because women tolerate pain better than men
    - Yes, thanks to a personalized approach based on the knowledge of the psycho-social context and of the patient's cultural derivation
    - No, the prognosis depends exclusively on biological healing and the natural history of the pathology

13. Could a correct application of Physiotherapy and Gender Medicine positively affect the economic sustainability of treatments by the Italian National Health System (INHS)?
    - No, indeed it would increase the cost by the INHS
    - No, I don't see how it could affect the economic sustainability of the INHS
    - Yes, ensuring faster and faster healing times
    - Yes, ensuring greater appropriateness of the treatment path
14. Is it important that the physiotherapist is trained in gender medicine and gender difference?
    - Yes, I consider it very important
    - Yes, I think it is important on average
    - Yes, I consider it sufficiently important
    - I don't think it's imported
15. Have you ever participated in training courses or information events on Gender Medicine?
    - Yes, more than once
    - Yes, only once
    - Never
16. Have you ever participated in training courses or information events on Gender Physiotherapy?
    - Yes, more than once
    - Yes, only once
    - Never
17. Do you apply the principles of gender medicine in your daily physiotherapy clinical activity?
    - Yes, always
    - Yes, but only with women
    - Yes, but only with men
    - Never
18. Do you think that your profession needs courses or events to acquire more knowledge on Gender Medicine?
    - Yes, I think it is necessary to deepen the knowledge in the field of Gender Medicine through courses and training events
    - No, I do not think it is necessary to deepen knowledge in the field of gender medicine through courses and training events
